# Potential of Guidewire‐Clot Interaction Forces for Clot Detection in Robotic Mechanical Thrombectomy

**DOI:** 10.1002/rcs.70109

**Published:** 2025-09-26

**Authors:** Evgenia Roussinova, Olivier Brina, William Galand, Philippe Reymond, Silvestro Micera, Paolo Machi, Mohamed Bouri

**Affiliations:** ^1^ REHAssist Group Neuro‐X Institute EPFL Lausanne Switzerland; ^2^ Division of Neuroradiology Geneva University Hospitals Geneva Switzerland; ^3^ Translational Neural Engineering Laboratory (TNE) Neuro‐X Institute EPFL Lausanne Switzerland; ^4^ Biorobotics Institute and Department of Excellence in Robotics and AI Scuola Superiore Sant’Anna Pisa Italy

**Keywords:** clot detection, guidewire, interaction forces, ischaemic stroke, robotic device

## Abstract

**Background:**

Interventional neuroradiologists currently miss crucial information when facing an ischaemic stroke case as they cannot localise the thrombus precisely nor determine its mechanical properties, which are both important for the clot extraction procedure.

**Methods:**

In this in vitro study, clot analogues of different stiffnesses and dimensions were inserted in an idealised arterial phantom using distinct insertion pressure levels. Guidewire‐clot interaction forces were recorded using a robotic device equipped with a force sensor. A model‐based clot detection method was developed.

**Results:**

Clot stiffness, initial clot volume, and insertion pressure were all found to impact the guidewire‐clot interaction forces. In detecting the clot's proximal and distal edges, we achieved success rates of 86.5% and 71.2%, respectively, within a tolerance of ± 1.5 mm.

**Conclusions:**

The study brings new perspectives for mechanical thrombectomy by demonstrating that multiple parameters influence the occlusion's mechanical state and by proposing algorithms for identifying the clot's extremities.

**Abbreviations**
ICA: internal carotid artery
MCA: middle cerebral artery
MTB: mechanical thrombectomy
RBC: red blood cells

## 1 Introduction

Stroke has been one of the leading causes of death worldwide for the last 20 years, with millions of people affected every year [1, 2]. Ischaemic stroke, responsible for over 3.5 million deaths annually and leaving many more with a severe disability [2], is a condition in which a blood clot occludes a cerebral artery. Currently, the standard treatment, especially for large vessel occlusions, is a minimally invasive endovascular procedure named mechanical thrombectomy (MTB), which consists of thrombus extraction through aspiration, stent retrieval, or a combined technique [3, 4, 5]. In the procedure, a thin flexible metallic wire (‘guidewire’) is used to navigate a series of catheters in a coaxial configuration from the femoral (or radial) artery up to the brain occlusion. First, a guiding catheter is advanced up to the level of the neck. Then, a microcatheter is navigated further to the location of the thrombus. In the case of aspiration, an additional catheter (an aspiration catheter) is placed close to the proximal edge of the thrombus. It is then connected to a pump and the clot is aspirated. In the case of the stent retrieval technique, a stent is deployed accross the clot, after which it is pulled out of the body, ideally with the clot attached to it. In order for the stent to be deployed, first, the guidewire penetrates the clot. Then, the microcatheter follows in crossing the lesion. Finally, the guidewire is replaced by the stent and the stent is deployed by retracting the microcatheter. Although the procedure has proved to be highly efficient in recent years, there are still critical issues leading to either a long procedure time, and therefore potentially a poor clinical outcome, or even a complete recanalisation failure [6, 7, 8].

One of the issues is inherent to the imaging technology used during the procedure, that is, digital subtraction angiography. Vessels are visualised by injecting a radiopaque contrast agent and taking biplanar x‐ray images. However, given that the clot obstructs the artery, neither its distal end nor the distal arterial tree can be visualised. In addition, in case of a layer of non‐contrasted blood just before the clot, the proximal extremity of the thrombus might not be precisely identified either. In order to improve the success rate of thrombectomy and reduce the procedure time, the operator needs to be able to localise the clot in the arterial tree. In the case of multiple branches at the level of the lesion, it is crucial to understand in which branch the clot is primarily located so that the extraction is performed at the correct location [9] as, otherwise, critical time can be wasted on trying to recanalise non‐occluded branches. Moreover, the location can also impact the choice of extraction technique. Bernava et al. [10] have shown that the angle between the main artery and the occluded branch is important for choosing between aspiration and stent retrieval as MTB techniques. Additionally, if a stent retriever is to be used, given that the stent should be deployed at the correct position, determining the proximal and distal ends of the clot is important, even when it is not located at a bifurcation. In this study, we developed methods for identifying the extremities of the thrombus based on the changes in the force signal acquired at the proximal end of the guidewire before, during, and after interaction with the clot.

Another issue decreasing the success of acute ischaemic stroke treatment is that operators are currently unable to determine the mechanical properties of the thrombus. A few groups have worked on the identification of the composition of the clot [11, 12, 13, 14]. Skyrman et al. [11, 12] have proposed an optical method, employing diffuse reflectance spectroscopy to differentiate between three types of clots—red blood cell (RBC)‐rich, fibrin‐rich, and mixed. The company Sensome (Massy, France) has developed a proprietary guidewire with an impedance sensor able to also distinguish between three clot categories—red (RBC‐rich), white (with low RBC content), and mixed [13, 14]. Although the results from these studies contribute to the overall understanding of the biological nature of the occlusion, since the clot removal is mechanical, we believe that a direct measurement of the mechanical properties of the clot in the artery is a missing key component.

In the past, groups have suggested the importance of the clot's stiffness, composition (which influences the stiffness) and size for the success of the extraction [10, 15, 16, 17, 18]. Typically, large stiff clots have been found to be more difficult to extract [10, 15]. RBC‐rich clots (usually softer than fibrin‐rich clots) have been found to be easier to extract [16, 17] especially with the aspiration technique [10, 18].

In a recent study [19], we showed in an anatomical arterial model that through a force measurement at the proximal end of the guidewire, stiff clots can be distinguished from soft clots based on several interaction force features. We also reported the qualitative observation that through the shape of the recorded force profiles during guidewire advancement through the clot, the proximal and the distal edge of the clot could be identified. This previous work served as a first proof of the utility of a force measurement combined with a precisely controlled guidewire displacement via a robotic device in an arterial model with a complex geometry. In this study, we reduce the impact of the geometry by choosing a simple arterial model having the shape of a tapered cylinder with the objective to focus on several factors which we hypothesised to influence the interaction forces between the guidewire and the clot, namely the stiffness and the initial dimensions of the clot, and the pressure with which the clot is pushed in the artery. Additionally, we formalised algorithms for the detection of the start and the end of the clot (i.e., the proximal and the distal edges).

## 2 Materials and Methods

### 2.1 Ethics Statement

Ethical approval was not required for this study as it did not involve human subjects or animals.

### 2.2 Experimental Setup

**FIGURE 1:**
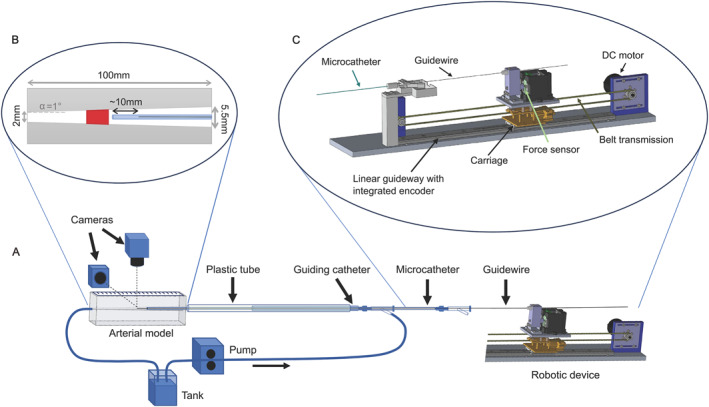
Experimental setup: (A) Overview of the experimental setup including a robotic device controlling the movement of the guidewire, a tapered cylinder arterial model made of silicone, a microcatheter and a guiding catheter for realistic support of the guidewire, a plastic tube simulating the arteries, a pump and tubing for perfusing the model, and two cameras capturing a top and a side view during the measurements. (B) Zoom on the dimensions of the arterial model and illustration of the configuration before each experimental run—catheter (in blue) placed approximately 1 mm away from the clot (in red), guidewire placed approximately 10 mm in the catheter. (C) Robotic device with one translational degree of freedom. The guidewire is mounted on a motorised carriage equipped with a force sensor.

The experimental setup is presented in Figure 1A. A straight 0.014 inches (0.36 mm diameter) guidewire (Avigo, Medtronic, Irvine, CA, USA) is fixed at its proximal end on a custom robotic device. The guidewire is inserted in a 0.021 inches (0.53 mm inner diameter) microcatheter (Phenom 021, Medtronic, Irvine, CA, USA) which itself is placed in a guiding catheter (Vista Brite Tip, Cordis, Hialeah, FL, USA). The two catheters are present to provide realistic support to the guidewire. The distal ends of the endovascular tools (guidewire and microcatheter) lie in the silicone arterial model. For this study, the arterial model was chosen to have a tapered cylinder shape. A pump (HBPIE‐24‐50‐400‐ZB‐2‐P, Dongguan Honlite Industrial Co. Ltd., Dongguan City, Guangdong Province, China) was used to perfuse the system with water before the experiments. Two cameras (VCXU‐124C, Baumer GmbH, Friedberg, Germany) were employed to capture two perpendicular views (top and side) at 10 frames/s during each measurement. A custom C++ programme was developed to control the robot, collect data from the system and synchronise the cameras.

### 2.3 Instrumented Robotic Device

The robotic device, shown in Figure 1C, is a one‐degree‐of‐freedom (1‐DoF) translational device. Its purpose is to control the movement of the guidewire and measure the axial forces exerted on it. The movement is realised through a direct current (DC) motor actuation with a belt transmission and guided by a linear guideway. The chosen force sensor is the MilliNewton‐B‐1000‐L‐U‐B (RYCONS Sensor Technology GmbH, Morges, Switzerland). The force signal is recorded at a sampling frequency of 1 kHz.

### 2.4 Idealised Arterial Phantom

The simplified arterial model (Elastrat Sàrl, Geneva, Switzerland) consists of a block of stiff silicone with a tapered cylinder opening in the middle. Silicone was selected as material by taking inspiration from the tests required by certifying agencies, such as the FDA (USA), for the approval of new endovascular tools (e.g., stents, catheters, guidewires). A section with key dimensions reported is presented in Figure 1B. The tapered cylinder is 100 mm long, with a taper angle of 1° and a distal diameter of 2 mm. The tapered hole was designed to cover the average diameters of the most commonly occluded large vessels in acute ischaemic stroke—the internal carotid artery (ICA) with a mean diameter ranging from 5 to 3.6 mm and the middle cerebral artery (MCA) with its portions M1 and M2 having average origin diameters between 3.1 and 2.4 mm, respectively [20, 21]. The taper angle was also chosen to be anatomically relevant given an average ICA taper of 0.04 mm/1 mm and an average M1 taper of 0.03 mm/1 mm, with a taper in our model of 0.35 mm/1 mm [21].

### 2.5 Fabrication and Characterisation of Synthetic Clot Analogues

**FIGURE 2:**
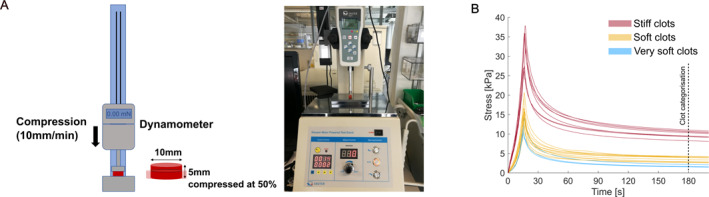
(A) Left: Illustration of the setup for clot characterisation. Right: Photo of the setup. (B) Clot compression curves from the characterisation procedure. The dashed line shows the moment at which the stress value was taken to categorise the clots.

Synthetic clots were prepared as hydrogels of guar gum and borax as reported in previous in vitro studies on ischaemic stroke [10, 19]. 3 g of guar gum were progressively incorporated in 100 mL of water using a magnetic stirrer. Then 1.5 g borax were added to the mixture. Finally, the mixture was stored in a closed container at room temperature. Over time, the mixture hardened and resulted in a clot‐like substance from which small cylindrical samples could be cut with a mechanical hole punch of a given diameter. A scalpel was used to trim the cylindrical sample to a desired length. Before each experiment, the mixture was characterised through an unconfined compression test as illustrated in Figure 2. A sample measuring 10 mm in diameter and 5 mm in height was compressed at a rate of 10 mm/min until 50% strain. Then the strain was kept constant for several minutes. During the test, the compression force was measured with a dynamometer (Sauter FL 10, Sauter GmbH, Wutöschingen, Germany). The compression curves in Figure 2B show a strain‐stiffening behaviour comparable to the one reported by Boodt et al. [22] for blood clots retrieved with thrombectomy from patients with acute ischaemic stroke. In addition, our synthetic clots present a stress‐relaxation behaviour also typical for real blood clots [23]. We used the stress value at 3 min from the start of the compression to categorise the clots as ‘very soft’, ‘soft’, and ‘stiff’. The corresponding ranges of stress at 50% strain after relaxation for the three categories were defined as follows: less than 2 kPa; from 2.5 to 4.5 kPa; more than 8 kPa.

### 2.6 Experimental Procedure

For each occlusion condition, two measurements were performed: a forward/penetration measurement, and a backward measurement. The initial configuration before the forward measurement is presented in Figure 1B. The clot analogue is inserted with a syringe, the pressure being measured with a pressure sensor (PSE573‐02, SMC, Tokyo, Japan). The experimenter applies the desired pressure (e.g., 120 mmHg) three consecutive times so that the clot reaches a stable position. The microcatheter is then placed approximately 1 mm away from the proximal edge of the clot. The distal end of the guidewire is positioned approximately 10 mm inside the catheter in order to measure a baseline force. The movement is then initiated and the guidewire is moved forward at 1 mm/s while penetrating the clot. The baseline is composed of the friction between the guidewire and the microcatheter occurring in the path before the clot. Its measurement serves to extract the interaction force between the guidewire and the clot from the total recorded force. The movement is stopped at around 10–15 mm after the end of the clot. Then, the backward measurement is performed by retrieving the guidewire again at 1 mm/s. In addition to the occlusion conditions, three measurements without clot were done for each experimental session, to be used as controls.

### 2.7 Investigation of the Impact of Clot Stiffness, Clot Dimensions, and Clot Insertion Pressure on the Clot‐Guidewire Interaction Forces

We conducted several experiments investigating the influence of three occlusion parameters—clot stiffness, clot size, and insertion pressure:

1. *Influence of the stiffness and the dimensions of the clot*
  For this set of experiments, the insertion pressure was kept at 120 mmHg. We varied the stiffness of the clot and the initial dimensions in order to obtain the same lodged position in the artery.
  1.1 First, we compared three types of clots (stiff, soft, and very soft). To reach the same position, the initial diameter was varied—7 mm for the very soft clots, 6 mm for the soft, and finally, 4 mm for the stiff ones. The initial length was kept constant—5 mm.
  1.2 In a second experiment, we varied both the initial diameter and the length of the clots in order to have the same position in the model but also the same quantity of material (i.e., initial volume). We compared clots with two stiffnesses—stiff and soft.
2. *Influence of the insertion pressure*
  In this experiment, stiff clots were injected at a lower pressure (60 mmHg), while soft clots were injected at a higher pressure (120 mmHg). The initial dimensions of the clots were selected so that the clots reached the same position in the arterial model. They were as follows: 5×6 mm for the soft clots and 3.5×9 mm for the stiff clots where dimensions are given in the format ‘diameter’×‘length’. The two pressure levels were chosen as corresponding to low and high mean arterial pressures of patients with stroke [24].
  A summary of the parameter values for the three experiments is presented in Table 1.

**TABLE 1:** Summary of the experimental parameters for the experiments investigating the impact of clot stiffness, clot size, and insertion pressure.

		Clot type	Clot compression value [kPa]	Initial D [mm]	Initial L [mm]	Insertion pressure [mmHg]
Exp. 1.1		Very soft	1.78	7	5	120
		Soft	3.13	6	5	
		Stiff	8.79	4	5	
Exp. 1.2	Session 1	Soft	3.44	6	4	120
		Stiff	10.78	3.5	11	
	Session 2	Soft	4.07	6	4	
		Stiff	10.51	4	8	
Exp. 2		Soft	4.12	5	6	120
		Stiff	9.42	3.5	9	60

*Note:* D stands for diameter. L stands for length.

### 2.8 Data Analysis

1. *Extraction of Start/End clot events*
  The timings of when the guidewire touches the proximal extremity of the clot and when the tip of the guidewire aligns with the distal extremity of the clot were extracted from the recorded images and named True Start and True End, respectively.
2. *Preprocessing of the force sensor voltage*
  The force sensor voltage was processed with a moving average filter with a window of 100 ms. It was then converted into a force in mN through the calibration curve of the force sensing unit, which is linear in the range of obtained forces.
3. *Average forward/backward force profiles*
  The force signals were cut in the region of interest: 5 s before and *T* seconds after the True Start (for forward)/True End (for backward), where *T* is different for each group of measurements depending on the length of the clot for the given experimental condition. Then the baseline of each cropped signal was removed so that the signal started around 0 mN. Finally, the mean and the standard deviation of the set of curves were calculated and plotted with an appropriate *x* axis ranging from −5 s to *T* seconds.
4. *Maximum force in clot*
  The maximum of the force signal between the True Start and True End was extracted and the baseline force in the catheter was subtracted to obtain the final value suitable for comparison between conditions.
5. *Maximum force slope in the beginning of penetration*
  The force signal was filtered with a 3rd order lowpass Butterworth filter with a cutoff frequency of 5 Hz. Then the slope was calculated and the maximum value in the first 3 seconds after the True Start was recorded.
6. *Delta force*
  The delta force was defined as the difference between the average force level beyond the clot and the baseline level before the clot. For the forward measurements, the level beyond the clot was taken once the force converged to a plateau.
7. *Average force slope in clot for backward measurement*
  The force signal was filtered with a 3rd order lowpass Butterworth filter with a cutoff frequency of 0.5 Hz. The slope was then calculated and the average value between the True End and True Start was reported.
8. *Calculating the volume of the clot in the arterial model*
  We used a reference ruler placed on top of the arterial mock‐up to calculate the radii of the truncated cone at the proximal $\left(R_{\text{prox}}\right)$ and distal $\left(R_{\text{dist}}\right)$ edges of the clot, as well as the length of the clot in the truncated cone right after insertion $\left(L_{\text{in}}\right)$. With these three parameters, we calculated the volume of the clot in the artery $\left(V_{\text{in}}\right)$ using the formula for a truncated cone: $V_{\text{in}}=\frac{1}{3}\pi L_{\text{in}}\left(R_{\text{prox}}^{2}+R_{\text{prox}}R_{\text{dist}}+R_{\text{dist}}^{2}\right)$

### 2.9 Statistics

The statistical significance of the difference between two groups was evaluated with the non‐parametric Wilcoxon rank sum test for unpaired samples. When more than two groups were compared, the non‐parametric Kruskal–Wallis test with Bonferroni correction was used.

### 2.10 Start/End Detection

The detection of the extremities of the clot that we propose is based on curve fitting on the force signal using a constrained non‐linear multivariable function optimisation; two different models are used for the forward and the backward measurements. The two events—Detected Start and Detected End—are calculated from the parameters of the models.

#### 2.10.1 Forward Model

For the model of the force during clot penetration, we took inspiration from studies on needle insertion in soft solids [25, 26, 27, 28, 29, 30]. Fregonese and Bacca [29, 30] have proposed a model based on accumulated theoretical and experimental knowledge from the past [26, 27, 28]. They formulated soft solid penetration as a two‐stage process—a first phase of indentation, comprising elastic deformation of the soft material before the indenter penetrates the surface, and a second phase of penetration, involving crack propagation, material separation, and constant dissipative work, with a sudden transition between the two phases characterised by a critical threshold.

Based on these previous works, as well as observations made during this study, the force signal obtained during the penetration of the clot was decomposed into several parts:

1. A plateau at the baseline until the Detected Start event $\left(f_{0}\right)$ reflects the friction between the guidewire and the catheter.
2. A polynomial increase in the force up to second degree upon contact with the clot $\left(f_{i}\left(t\right)\right)$ allows us to model two physical phenomena—the clot indentation phase as described by [29] and the possible buckling of the guidewire, which is a phenomenon observed during our study, with more detail provided in the Results section.
3. A drop in the force representing the transition between the first and second phases of penetration described above, modelled with a smooth discharge of the energy accumulated in buckling during the first phase of indentation $\left(f_{relax\text{\_}i}\left(t\right)\right)$. Here we opt for a simple function accounting for the observed behaviour without providing a full analytical model of the buckling phenomenon.
4. A linear increase in the force in the clot during the penetration phase $\left(f_{p}\left(t\right)\right)$.
5. A smooth discharge of the energy accumulated in buckling in the clot $\left(f_{relax\text{\_}p}\left(t\right)\right)$.
6. A plateau beyond the clot $\left(f_{\text{end}}\right)$.

**FIGURE 3:**
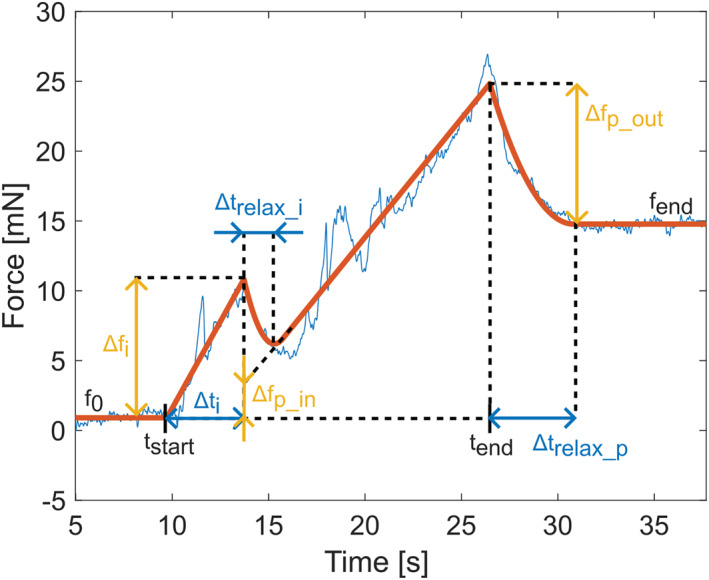
An example of a model fit for the forward measurement with key parameters illustrated. In blue is the measured force with removed offset. In red is the fitted model curve.

An example of a forward model fit with the corresponding parameters is shown in Figure 3. The formal definition of the model is given by the following equations:

(1) $$\begin{array}{c} F(t)=\left\{\begin{array}{cc} f_{0},\; & t_{0}\leq t\leq t_{\text{start}}\\ f_{0}+f_{i}(t),\; & t_{\text{start}}<t\leq t_{\text{start}}+\Delta t_{i}\\ f_{0}+f_{\mathrm{relax}\text{\_}i}(t)+f_{p}(t),\; & t_{\text{start}}+\Delta t_{i}<t\leq t_{\text{end}}\\ f_{\text{end}}+f_{\mathrm{relax}\text{\_}p}(t),\; & t>t_{\text{end}} \end{array}\right. \end{array}$$

$$f_{i}\left(t\right)=\Delta f_{i}{\left(\frac{t-t_{\text{start}}}{\Delta t_{i}}\right)}^{k},\;k\in \left[1,2\right]$$

$$f_{p}\left(t\right)=\Delta f_{p\text{\_}in}+\left(f_{\text{end}}+\Delta f_{p\text{\_}out}-\left(f_{0}+\Delta f_{p\text{\_}in}\right)\right)\cdot \frac{t-\left(t_{\text{start}}+\Delta t_{i}\right)}{t_{\text{end}}-\left(t_{\text{start}}+\Delta t_{i}\right)}$$

$$f_{relax\text{\_}i}\left(t\right)=\left(\Delta f_{i}-\Delta f_{p\text{\_}in}\right)\cdot {\left(\frac{t-\left(t_{\text{start}}+\Delta t_{i}+\Delta t_{relax\text{\_}i}\right)}{\Delta t_{relax\text{\_}i}}\right)}^{2}$$

$$f_{relax\text{\_}p}\left(t\right)=\Delta f_{p\text{\_}out}{\left(\frac{t-\left(t_{\text{end}}+\Delta t_{relax\text{\_}p}\right)}{\Delta t_{relax\text{\_}p}}\right)}^{2}$$

For the forward model, $t_{\text{start}}$ corresponds to $t_{start\text{\_}clot}$. However, due to the flexibility of the guidewire and the elasticity of the clot, $t_{\text{end}}$ does not necessarily correspond to $t_{end\text{\_}clot}$. We found that $t_{end\text{\_}clot}$ is often between $t_{\text{end}}$ and the start of the plateau, observed beyond the clot. In order to decide where exactly to define the Detected End event with respect to the optimised model parameters, we trained an additional parameter r and found that the best value for the considered dataset is 0.13. Therefore, the Detected End $\left(t_{end\text{\_}clot}\right)$ was defined as follows:

$$\begin{array}{l} t_{end\text{\_}clot}=t_{\text{end}}+r\Delta t_{relax\text{\_}p}\\ =t_{\text{end}}+0.13\Delta t_{relax\text{\_}p} \end{array}$$

#### 2.10.2 Backward Model

The backward force signal was fitted with a simple curve consisting of a constant slope $\left(s_{1}\right)$ beyond the clot, another slope $\left(s_{2}\right)$ within the clot and a plateau after the Detected Start. The curve is defined by the following equations:

(2) $$F\left(t\right)=\left\{\begin{array}{cc} f_{1}+s_{1}\left(t-t_{0}\right),\; & t_{0}\leq t\leq t_{end\text{\_}clot}\\ f_{2}+s_{2}\left(t-t_{end\text{\_}clot}\right),\; & t_{end\text{\_}clot}<t\leq t_{start\text{\_}clot}\\ f_{3},\; & t>t_{start\text{\_}clot} \end{array}\right.$$

with $s_{1}=\frac{f_{2}-f_{1}}{t_{end\text{\_}clot}-t_{0}}$ and $s_{2}=\frac{f_{3}-f_{2}}{t_{start\text{\_}clot}-t_{end\text{\_}clot}}$

**FIGURE 4:**
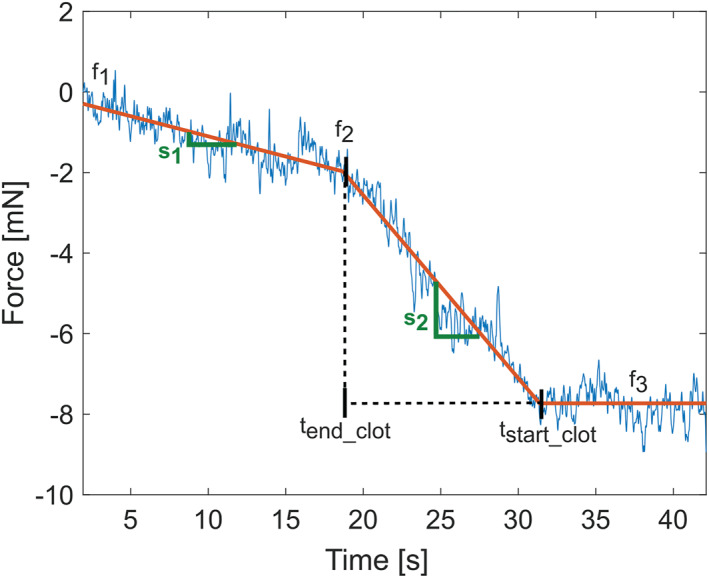
An example of a model fit for the backward measurement with key parameters illustrated. In blue is the measured force with removed offset. In red is the fitted model curve.

An example of a backward model fit with the corresponding parameters is shown in Figure 4.

#### 2.10.3 Duration of Model Fits

We measured the time it takes to fit each model (forward and backward) by using the MATLAB R2023a (MathWorks Inc., Natick, MA, USA) integrated stopwatch timer. The goal was to evaluate to which extent the proposed detection methods are potentially applicable in the clinical procedure.

### 2.11 Clot Length Estimation

For each trial, we performed the Start and End detection as described in the previous section. We then evaluated the estimation of the length of the clot by combining the results from the detection. The result is a detected interval, which is to be compared with the real interval between True Start and True End.

### 2.12 Extraction of the Guidewire Tip Positions for True and Detected Start and End

In order to evaluate the detection performance in space, we developed a semi‐automated method for extracting the position of the tip of the guidewire in millimetres for key events. To do so, first, the guidewire was segmented through an image processing algorithm. Second, the tip was identified as the utmost left pixel of the segmented line. In order to transform the pixel location into a position in mm, the ruler, present on each image, was detected through another image processing algorithm and the distances between the ruler lines were used for the calculation of the pixel‐to‐mm ratio. Finally, the automatically generated results were reviewed by a human. For trials with incorrect detection of the tip, the researcher would use a graphical user interface (GUI) to click on the tip and record its position. For trials with poor ruler detection, the researcher would use another GUI to click on two scale lines 10 mm apart in order for the pixel‐to‐mm ratio to be calculated.

## 3 Results

### 3.1 General Force Profiles

The first results of the study concern the shape of the force profiles observed during clot penetration as well as during guidewire retrieval.

**FIGURE 5:**
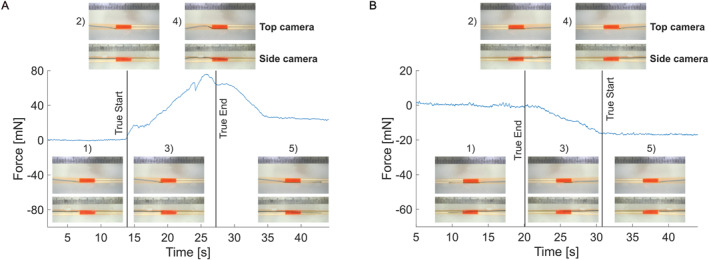
Example of recorded forward and backward force profiles for an experimental run with images from the two cameras illustrating the different phases of the measurement. (A) Forward measurement: (1) advancement in the catheter (baseline); (2) contact with the clot; (3) penetration of the clot (in this case between the clot and the wall); (4) buckling of the guidewire (possible at contact or as illustrated here in the clot); (5) guidewire beyond the clot (plateau in the force). (B) Backward measurement: (1) guidewire beyond the clot; (2) guidewire tip aligned with the distal edge of the clot (True End label); (3) guidewire within the clot (progressive decrease in the force level); (4) guidewire tip aligned with the proximal edge of the clot (True Start label); (5) guidewire about to be retrieved in the catheter.

*During penetration* the guidewire either needs to pierce the clot, similar to a needle penetrating soft tissue, or separate the clot from the arterial wall. It must then provide enough force to propagate the crack and overcome the friction during the advancement. Given the flexibility of the guidewire, it is prone to buckling under the axial force either upon contact with the clot before the penetration, or in the clot during the penetration. The buckling results in an accumulation of elastic energy and eventually its release, which can be seen as a progressive drop in the recorded force. Once the guidewire's tip is out of the clot, the main force acting on it is constant friction (when the viscous force with the liquid outside the clot is negligible), so the recorded signal presents a plateau. The plateau does not necessarily come right at the end of the clot and is not necessarily the peak of the force, depending on the occlusion conditions (e.g., stiffness of the clot, at which pressure the clot is inserted). An example is shown in Figure 5A.

In our experiments, the movement of the guidewire starts in the catheter where only friction is present, which is confirmed by the flatness of the force profile before contact with the clot.

*During the retrieval/backward measurement* the main particularity is that there is no buckling of the guidewire, given that it is being pulled and not pushed, and only the friction between the guidewire and the clot and/or the arterial wall is measured. This results in a force profile with a plateau or a slight negative slope beyond the clot, gradual decrease of the force in the clot and a plateau when the guidewire enters the catheter again. An example is shown in Figure 5B. The slope beyond the clot can happen if the guidewire slides against the artery and during the retrieval the contact surface between the two decreases, thus the friction decreases.

### 3.2 Occlusion Parameters

In the next sections, we investigated how the force profiles depend on the occlusion parameters and how we can rely on the measured force to identify the extremities of the clot. In terms of parameters, we focused on the following: clot stiffness, dimensions, and insertion pressure. We were particularly interested in comparing different occlusion conditions that can be encountered at the same arterial position.

#### 3.2.1 Clots With Different Stiffnesses Ending up at the Same Position in the Artery—Influence of the Stiffness and the Dimensions of the Clot

**FIGURE 6:**
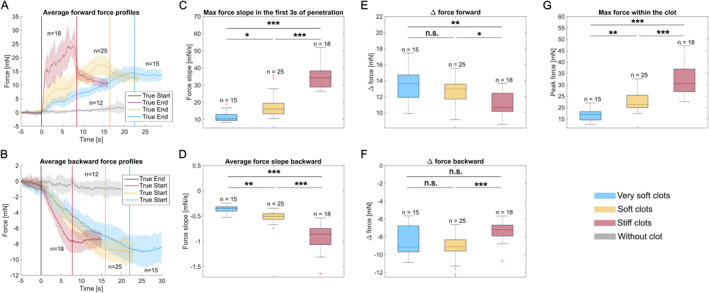
Interaction forces between a guidewire and clots with different stiffnesses, blocked at the same location in the arterial model. Insertion pressure was 120 mmHg. The initial length of all clots was 5 mm and the initial diameters were 7, 6, and 4 mm for very soft, soft, and stiff clots, respectively. For the soft clots category, two sessions with *n* = 15 and *n* = 10 samples were combined. (A) Average force profiles for the forward measurement. (B) Average force profiles for the backward measurement. (C) Boxplots of the maximum force slope in the first 3 seconds after the True Start. (D) Boxplots of the average force slope backward. (E) Boxplots of the delta force for the forward measurement. (F) Boxplots of the delta force for the backward measurement. (G) Boxplots of the peak force within the clot; *p <0.05, **p <0.01, and ***p <0.001.

Figure 6 presents the results from the experiment in which three types of clots were compared—stiff, soft, and very soft. Panels A and B show the obtained average curves for the forward and backward measurements. Given the differences in the initial volumes, the lengths in the model for the three types of clots are also different, namely the very soft is the longest (∼20 mm) and the stiff is the shortest (∼7 mm). This can be observed on the plots through the timing of the True End events. Another difference observed between the three types of clots is the force slope in the beginning of penetration, as well as the maximum force within the clot; they are both higher for the stiffer clots (panels C and G). Similarly, the slope in the clot during the backward measurement is the most negative for the stiff clots, followed by the soft clots and finally the very soft clots (panel D). On the other hand, the plateau forces, reflecting the total friction with the clot, tend to reach similar values or even higher for the soft clots (panels E and F).

**FIGURE 7:**
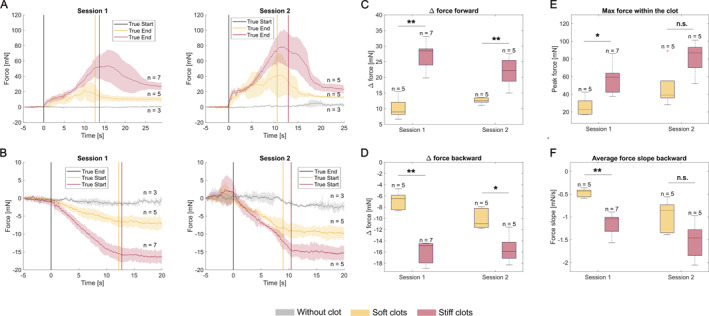
Interaction forces between a guidewire and clots with different stiffness and approximately the same initial volume blocked at the same location in the arterial model. Insertion pressure was 120 mmHg. (A) Average force profiles for the forward measurement. (B) Average force profiles for the backward measurement. (C) Boxplots of the delta force for the forward measurement. (D) Boxplots of the delta force for the backward measurement. (E) Boxplots of the peak force within the clot. (F) Boxplots of the average force slope backward; *p <0.05, **p <0.01, and ***p <0.001.

Our hypothesis for the observed close values for the plateau (delta force) was that the difference in the initial volume of the clots played an important role. To verify this, we did a second experiment in which we adjusted both the initial diameter and the length of clots with two stiffnesses in order to have the same position in the arterial model but also the same initial clot volume, similar to what was done in our previous study in an anatomical arterial model [19]. The results are presented in Figure 7. In this case, it can be observed the plateaus are clearly separated, both in the forward and in the backward measurements, with the stiff clots generating higher forces than soft clots at volume parity (panels A–D). This was observed in two sessions with two sets of stiff/soft clots. The peak force within the clot was also observed to be in general higher for the stiff clots than for the soft ones, consistent with the results from [19], with a statistically significant difference obtained for one of the sessions (panel E). Surprisingly, the slopes in the forward measurement were very similar and no statistical significance was found for the maximum slope in the beginning of penetration (*p* = 1 for Session 1 and *p* = 0.42 for Session 2). However, in the backward measurement the average slope in the clot was in general more negative for the stiffer clots than for the softer clots, with a statistically significant difference for one of the sessions (panel F). Even though in the previous experiment the slope in the beginning of penetration was a distinguishing factor between the three types of clots, it appears that it is not a general rule. A soft clot can be as tough to pierce as a stiff clot. However, the total resistance on the guidewire, reflected by the plateau levels, was higher for the stiff clots.

#### 3.2.2 Influence of the Insertion Pressure—Stiffer Clots Inserted at 60 mmHg Versus. Softer Clots Inserted at 120 mmHg

**FIGURE 8:**
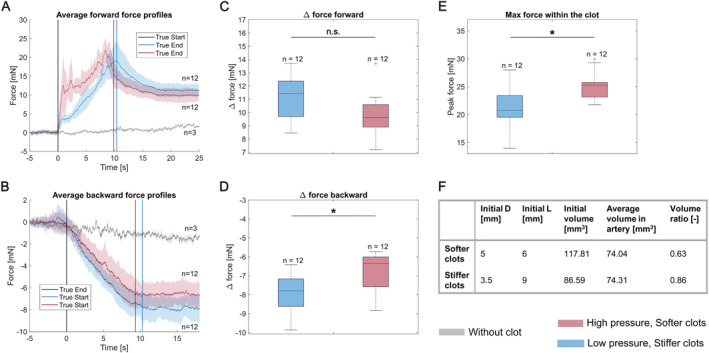
Interaction forces between a guidewire and clots with two different stiffnesses inserted at two different pressures. (A) Average force profiles for the forward measurement. (B) Average force profiles for the backward measurement. (C) Boxplots of the delta force for the forward measurement. (D) Boxplots of the delta force for the backward measurement. (E) Boxplots of the peak force within the clot. (F) Table summarising the initial dimensions of the clots as well as their volume in the arterial model; *p <0.05, **p <0.01, and ***p <0.001.

Apart from the dimensions and the stiffness of the clot, we hypothesised that the pressure with which the clots get inserted into the artery has an important impact on the mechanical properties of the occlusion and therefore on the interaction forces between the guidewire and the clot. In a third experiment, we injected stiffer clots at a lower pressure (60 mmHg) and softer clots at a higher pressure (120 mmHg) reaching the same position in the artery. The first observation to be made is that due to the different pressures, clots with different initial volumes ended up at the same position and with the same length in the arterial model (Figure 8, panel F). Indeed, when calculating the volume ratio, it appears that the softer clots were more compacted under the high pressure than the stiffer clots, which were inserted at a lower pressure. In terms of forces, it can be observed in panels A–E that the soft clots generated similar if not higher forces (e.g., the peak force) than the stiff clots. Therefore, the mechanical characteristics of the occlusion depend not only on the stiffness of the clot, but also on the insertion pressure. This is an important result because it shows that higher pressure can mask the softness of the clot and therefore it is a crucial factor to take into consideration when evaluating the mechanical properties of the occlusion with the aim of choosing an appropriate extraction strategy.

### 3.3 Start/End Detection

**FIGURE 9:**
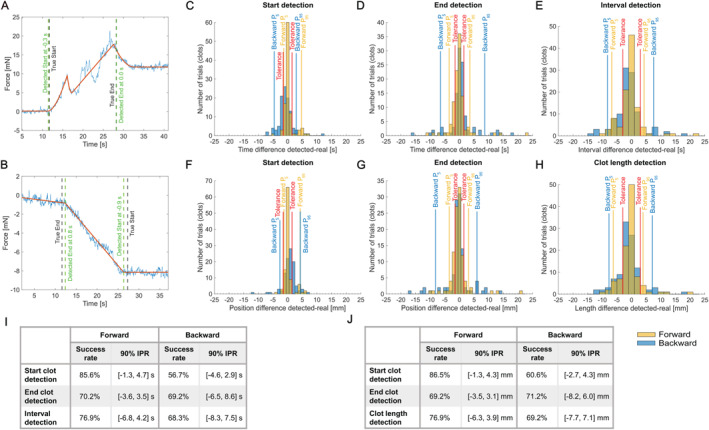
Detection of the extremities of the clot. (A) Example of model fit for the forward measurement. In blue, a recorded force profile for a clot penetration (forward) measurement. In red, the result from the optimisation process for the model fit. (B) Example of model fit for the backward measurement. In blue, a recorded force profile for a backward measurement. In red, the result from the optimisation process for the model fit. (C) Histograms of the time difference between the Detected Start and the True Start (visually determined from the recorded images) for the forward (in yellow) and backward (in blue) measurements. The red ‘Tolerance’ lines mark the $\left[-1.5,1.5\right]\;s$ interval considered as a successful range for the detection. The blue and yellow lines mark the 5th and 95th percentiles. (D) Histograms of the time difference between the Detected End and the True End. (E) Histograms illustrating the performance in estimating the length of the clot through the interval detection. The red ‘Tolerance’ lines mark the $\left[-3,3\right]\;s$ interval considered as a successful range for the detection. (F) Histograms of the detection error in millimetres between the Detected Start position and the True Start position. The red ‘Tolerance’ lines mark the $\left[-1.5,1.5\right]\;mm$ interval considered as a successful range for the detection. (G) Histograms of the detection error in millimetres between the Detected End position and the True End position. (H) Histograms illustrating the performance in detecting the length of the clot. The red ‘Tolerance’ lines mark the $\left[-3,3\right]\;mm$ interval considered as a successful range for the detection. (I) Table summarising the detection performance in time with the success rate and 90% interpercentile range (IPR). (J) Table summarising the detection performance in space.

The beginning and the end of the clot were detected through both forward and backward measurements. In Figure 9, panels A and B, examples of model fits are shown for the two measurements with the true events, as well as the detected events. Panels C and D present the distributions of the difference in time between the detected and the real event, respectively, for the Start and the End. Panels F and G present the distributions of the detection error in space. 104 trials were included in this analysis. This corresponds to all occlusion conditions presented here across the various figures. For the error on the Start detection, we can observe in panel C that the forward measurement gives a narrow peak around 0, which is desired. However, for a few trials we notice late detection around 5 s delta time. With the backward measurement, the Detected Start is more shifted towards earlier detection with a larger spread. Given the constant penetration speed of 1 mm/s, one second of delta time corresponds approximately to 1 mm detection error. This is an approximation since due to the flexibility of the guidewire, the displacement of its proximal end, which is precisely controlled by the robot, does not have a 1:1 correspondence with the displacement of the distal end. It can be observed in panels F and G, where the detection error in millimetres is shown, that the approximation is appropriate given the similarity with panels C and D, which show the detection error in time. Here it should be noted that for the backward measurement, a positive time difference, that is, a later detection, results in a negative position difference, that is, a more proximal detected position. We defined the success range as ±1.5 s in time and ±1.5 mm in space around the real event or position, respectively. The results in terms of success rate are presented in panels I and J. In time, we achieved 85.6% success with the forward measurement force signal and 56.7% success with the backward measurement for the Start detection. In space, the success rates were 86.5% and 60.6%, respectively. By calculating the 5th and 95th percentiles, we evaluated the 90% interpercentile ranges: $\left[-1.3,4.3\right]\;mm$ for the forward and $\left[-2.7,4.3\right]\;mm$ for the backward measurement. As for the End detection, in space we obtained 69.2% success with the forward model and 71.2% with the backward model. The 90% interpercentile ranges are $\left[-3.5,3.1\right]\;mm$ and $\left[-8.2,6.0\right]\;mm$ for forward and backward measurement, respectively. On the histograms shown in Figure 9G, one can see that for the forward measurement, the detection is again centred around 0 but with a larger spread than for the detection of the Start (panel F). With the backward measurement, a similar distribution is observed. Unlike the Start detection, where the forward model clearly outperformed, for the End detection, the backward measurement was as successful with a slightly higher success rate but a larger 90% interpercentile range.

In terms of computational time for each fit, we measured that on a laptop running Windows 11 OS, with a 1.8 GHz Intel Core i7 8th Gen CPU and 16 GB RAM, the forward fit takes on average 1.71 s with a standard deviation of 0.94 s, while a backward model fit takes 0.76 s with a standard deviation of 0.59 s. This is acceptable within the timeline of the procedure which takes tens of minutes.

When combining the Start and End detections to estimate the length of the clot, we obtained the results presented in Figure 9E. The success tolerance is ±3 s, which is the combined tolerance on the two events' detection. In panel H are presented the actual results of the clot length detection in millimetres with the tolerance of ±3 mm. With the method based on the forward measurement, we achieved a success rate of 76.9%, compared to 69.2% with the method based on the backward measurement.

The results reported above concern the whole dataset. To evaluate the robustness of the methods, we performed a 5‐fold cross‐validation repeated 40 times, the results from which are presented in Table 2. The mean success rates are in line with the results reported for the whole dataset. The smallest standard deviation (6.9%) is obtained for the Start detection with the forward measurement, showing the best robustness of the model fitting. On the other hand, the Start detection for the backward measurement has the worst robustness. It is coherent with the bad success rate observed previously and confirms that forward detection is preferable to backward detection for the start of the clot. It should be mentioned that with the forward method, we have a parameter which is trained (r). In the cross‐validation analysis, this parameter was trained on the corresponding training set. However, for the analysis presented in Figure 9, the parameter was trained on the whole dataset. Given that the manual verification procedure on the guidewire tip position identification would have required a huge effort (i.e., a check and a potential correction on more than 4000 images) while the results in time give already a good indication of the detection performance, the cross‐validation analysis was performed only in time and not in space.

**TABLE 2:** Summary of the results from the 5‐fold cross‐validation performed on the Start/End clot detection—mean and standard deviation of the detection success rate in time.

	Success rate	
	Forward	Backward
	Mean ± SD	Mean ± SD
Start detection	85.7%±6.9%	56.8%±9.6%
End detection	65.6%±9.5%	69.1%±9.2%
Interval detection	76.9%±8.8%	68.4%±9.5%

## 4 Discussion

### 4.1 Contributions

The present study addresses two important topics for mechanical thrombectomy. The first one is measuring the mechanical behaviour of the occlusion for different conditions, which has not been done so far. In particular, the importance of the insertion pressure is demonstrated through an example in which an initially soft clot appears to be stiffer in the artery because of a higher impaction force. It is an open question whether a soft clot compacted with a high pressure behaves similarly during thrombectomy as a stiff clot that was lodged at a lower pressure. The volume of the clot is also shown to influence the total friction force exerted on the guidewire, which we hypothesise to correlate with the force between the clot and the artery during extraction. Until now, most research in the field has been focused on identifying the composition of the clot or relating the success of extraction to the clot's composition. Even if the biochemical composition is related to the mechanical properties of the material [16, 17, 18, 31, 32], this study shows that there are other factors influencing the properties of the occlusion, and therefore an in situ force measurement may be necessary for a better estimation of the mechanical behaviour of the clot within the artery.

The second topic that we investigated was how to identify the extremities of the clot. For the proximal edge, interventionalists already have an idea through the angiography as the contrast agent stops at the level of the occlusion. However, if the contrast liquid does not manage to flow well up to the clot, the information from the imaging might not be completely accurate. As for the distal edge, to the best of our knowledge, there is currently no robust method for its identification. One technique evoked in the literature [33] is to cross the supposed location of the clot with the microcatheter and to inject contrast liquid beyond the clot. If the contrast liquid flows back to the distal edge of the clot, it could give an indication about the location of the clot. However, such a retrograde flow is not guaranteed depending on the collateral flow conditions, on the lumen of the artery compared to the diameter of the catheter, etc. In addition, crossing the clot with the catheter brings risks of fragmentation of the thrombus, which could lead to severe complications. Therefore, it may be better to rely only on the guidewire clot penetration as presented in this study. Another technique, recently reported as promising for the visualisation of the distal edge, is the contrast‐enhanced cone beam computed tomography (CE‐CBCT) [34]. This imaging method, however, relies largely on collateral circulation, which depends on the type of occlusion and remains highly subject‐dependent.

The approach proposed in this work relies on a force measurement at the proximal end of the guidewire. A primary challenge is the significant distance between the sensor and the clot, with a flexible element in between. This introduces variability coming from different phenomena such as bending and buckling, which are accounted for in our force models. The advantage of such an approach is that the guidewire does not need to be modified; therefore, first, it has the mechanical properties of guidewires currently used in the clinic, and second, the approach is more directly translatable to the clinical practice. Also, this force‐based method does not rely on the opacification of the vessels; it introduces a new source of information to improve the operator's comprehension of the occlusion.

### 4.2 Experimental Results

In terms of the results regarding the force profiles for different occlusion conditions, there were a few interesting observations. First, the slope in the beginning of penetration was not found to be a distinguishing factor between clots with different stiffnesses in all conditions. A possible explanation is that the toughness of the clot plays an important role, however, as explained in the ‘Limitations’ section, we did not have an appropriate setup to measure this property and therefore it is an unknown variable in the study. Second, we showed that the insertion pressure is an important factor of the occlusion. However, its exact influence is not completely understood as in our experiment it seems to have influenced more the maximum force, rather than the delta forces. In the future, its impact should be further investigated.

As for the results from the Start/End detection, the forward model resulted in a better performance than the backward one for proximal edge detection. This is reasonable as during the first phase of the penetration, the force usually increases rapidly compared to the gradual decrease for the backward movement. For the distal edge of the clot, we obtained a similar performance for the two models. The disadvantage of the forward model is that there is a parameter which is trained on a finite dataset. On the contrary, for the backward model, the Detected End is a deterministic parameter of the curve so it might be more stable in terms of generalisation. To make the forward model deterministic, an explicit definition of the currently trained parameter *r* would be necessary. Since *r* is intrinsically linked to the physical properties of both the guidewire and the clot—representing the release of elastic energy stored during clot penetration—we could infer its value by establishing a correlation between the parameter and specific features of the force profile.

### 4.3 Limitations

The main limitation of the study concerns the controlled variables that we used. We controlled for the stiffness and dimensions of the clot and the insertion pressure. We also controlled for the lodged position of the clot in the arterial mock‐up. However, we did not control for the friction coefficient between the clot and the guidewire or the artery, or for the toughness of the clot. Both properties are not trivial to measure given that the clot is a hydrogel (so with a high water content). Thus, the friction depends highly on the moisture of the testing surface and can get confounded with surface tension effects. On the other hand, the toughness requires a setup to clamp the clot and pull in opposite directions and measure the force, or the use of scissors instrumented with a force measurement to cut the clot [27]. Due to setup limitations, we could not measure these two properties and left them as unknowns.

A limitation regarding the analysis is that we did not have a direct measurement of the position of the tip of the guidewire. As mentioned earlier, given the flexibility of the guidewire, there is not a perfect match between proximal end and tip displacement. For the evaluation of the detection performance, we identified the position of the tip for the key events (detected and real Start and End). However, for the rest of the measurements' duration, we did not have the tools to perfectly track the tip. Therefore, it should be mentioned that we present the recorded force profiles in time, whereas in the studies of soft solid penetration, usually the curves are force as a function of the penetration depth. Therefore, despite the similarities between the profiles, one should not make a direct comparison between the curves.

### 4.4 Future Directions

For this study, we used an idealised arterial model, having the shape of a tapered cylinder, and a straight configuration up to the model. This is justified because the study's aim was to analyse the interaction forces during clot penetration for different occlusion conditions and therefore the first step was to understand the effects at play with a basic configuration. In the future, the findings of this study should be confirmed in more complex, anatomically relevant geometries. In this study, we used synthetic clot analogues, presenting several behaviours typical for real blood clots. However, the used synthetic material does not capture the whole complexity of real blood clots. Therefore, a validation with real thrombi, homogeneous as well as heterogeneous analogues, should be performed. In addition, we used a straight guidewire, whereas in the clinic, operators use a shaped guidewire with a certain angle (most commonly 45°). In order to obtain a formed guidewire, interventionalists usually shape it manually, which introduces an additional variability since every time the shape is slightly different. We opted for a straight guidewire as we wanted to specifically analyse the effect of the controlled parameters. In the future, the impact of the shape of the tip of the guidewire should be studied. The impact of potential difference in the mechanical properties of different models of guidewires should also be investigated. Our goal for the future is to understand quantitatively the contribution of each parameter to the force profile and to the success of different extraction strategies. With this, combined with the localisation of the clot through the detection of its extremities, we would be able to develop an approach to select an extraction technique based on a force measurement performed once, and thus hopefully reach first‐pass recanalisation. Finally, it has to be noted that we advanced the guidewire at 1 mm/s without taking care of any possible excess of energy accumulation during clot penetration and its sudden, possibly harmful release. Therefore, in the future, the safety of the penetration has to be carefully evaluated. In addition, the impact of velocity variabilities for designed robotic penetration profiles, or manual navigation, on the recorded force profiles should be studied.

## 5 Conclusions

In this work, we used a robotic device, designed for navigating a guidewire and measuring the axial forces sustained by it, to investigate the interaction forces between a 0.014 inches guidewire and synthetic clots with different stiffnesses and dimensions, inserted in an arterial mock‐up of tapered cylinder shape at a controlled pressure. We discovered that the clot penetration force profile is quite similar to the one observed during needle insertion into soft tissue, with the differences that guidewires are more prone to buckling, which adds a different component to the force curve. We showed with a few experiments that all three parameters mentioned above are key for the total friction in the occlusion. We also concluded that other material properties might be necessary to fully explain the measured forces and this requires further investigation. Finally, we proposed two methods for the identification of the clot's extremities based on fitting physics‐relevant model curves, which brings new perspectives for the treatment of acute ischaemic stroke. In the future, the findings on the interaction forces, as well as the detection methods, should be extended to anatomical geometries and clinically relevant guidewire shapes.

## Author Contributions

The authors confirm the contribution to the paper as follows: study conception: E.R., P.M., M.B.; study supervision: M.B., P.M., S.M.; data acquisition: E.R., O.B., P.R.; data analysis and interpretation of results: E.R., W.G.; figures preparation: E.R., O.B., W.G.; manuscript preparation: E.R., W.G. All authors reviewed the manuscript.

## Ethics Statement

The authors have nothing to report.

## Conflicts of Interest

The authors declare no conflicts of interest.

## Data Availability

The experimental data from this study are available upon reasonable request.
